# Errata

**DOI:** 10.4269/ajtmh.2010.10-1202e

**Published:** 2010-06

In *Am J Trop Med Hyg 82:* 838–845, the financial support section should have stated that Dr. Ruiz is a member of the Research Career of the National Council of Scientific and Technical Research (CONICET) from Argentina. The journal regrets this omission.

In *Am J Trop Med Hyg 66:* 605–610, one of the author's last names is misspelled. The correct spelling of the sixth author's name is Ippolytos Kalofonos. The journal regrets the error.

